# First-Principles Study of B_16_N_16_ Cluster-Assembled Porous Nanomaterials

**DOI:** 10.3390/nano13131927

**Published:** 2023-06-24

**Authors:** Xin Wang, Xiaoyue Zhang, Liwei Liu, Tielei Song, Zhifeng Liu, Xin Cui

**Affiliations:** School of Physical Science and Technology, Inner Mongolia University, Hohhot 010021, China; m15847608829@163.com (X.W.); 18347471082@163.com (X.Z.); l18648294612@126.com (L.L.); tlsong@imu.edu.cn (T.S.); zfliu@imu.edu.cn (Z.L.)

**Keywords:** boron nitride, cluster-assembled materials, mechanical properties, electronic properties, first principles calculations

## Abstract

Owing to the similar valence electron structures between the B-N bond and the C-C bond, boron nitride, similar to carbon, can form abundant polymorphs with different frameworks, which possess rich mechanical and electronic properties. Using the hollow, cage-like B_16_N_16_ cluster as building blocks, here, we established three new BN polymorphs with low-density porous structures, termed Cub-B_16_N_16_, Tet-B_16_N_16_, and Ort-B_16_N_16_, which have cubic ($P\bar{4}3m$), tetragonal (*P*4/*nbm*), and orthomorphic (*Imma*) symmetries, respectively. Our density functional theory (DFT) calculations indicated that the existence of porous structure Cub-B_16_N_16_, Tet-B_16_N_16_, and Ort-B_16_N_16_ were not only energetically, dynamically, thermally and mechanically stable, they were even more stable than some known phases, such as sc-B_12_N_12_ and Hp-BN. The obtained Pugh’s ratio showed that the Cub-B_16_N_16_ and Tet-B_16_N_16_ structures were brittle materials, but Ort-B_16_N_16_ was ductile. The analysis of ideal strength, Young’s moduli, and shear moduli revealed that the proposed new phases all exhibited sizable mechanical anisotropy. Additionally, the calculation of electronic band structures and density of states showed that they were all semiconducting with a wide, indirect band gap (~3 eV). The results obtained in this work not only identified three stable BN polymorphs, they also highlighted a bottom-up way to obtain the desired materials with the clusters serving as building blocks.

## 1. Introduction

The similar valance electronic configuration between the B-N bond and the C-C bond makes boron nitrides have many polymorphs, similar to carbon, including zero-dimensional (0D) clusters, one-dimensional (1D) nanotubes and nanoribbons, two-dimensional (2D) nanosheets, and three-dimensional (3D) crystalline or amorphous BN [1,2,3,4,5]. As a new level of material structure, the study of clusters is helpful to understand the evolution law of matter from microscopic atoms and molecules to macroscopic condensed matter; it also provides an ideal platform to explore the novel physic phenomena of the 0D nano-system.

In recent years, B*_k_*N*_k_* cage clusters have attracted numerous attentions, due to their potential application in materials, energy, environment, and other fields [6,7,8,9,10,11,12,13,14,15,16,17]. From previous works, one can know that there are two main types of B*_k_*N*_k_* cage structures: one is constructed from alternating B-N bonds and consists entirely of four-membered and six-membered rings. The other is a fullerene-like structure based on combinations of pentagons and hexagons, with N-N and B-B bonds [17,18,19]. Oku et al. [6,7,8,9] synthesized and detected B*_k_*N*_k_* (*k* = 12, 24–60) nanocages by laser desorption time-of-flight mass spectrometry and found that the BN clusters consisting of 4-, 6- and 8-membered BN rings satisfied the isolated tetragonal rule, which was optimized by molecular orbital calculation. Stéphan et al. [10] presented experimental evidence for the formation of small BN cage-like molecules by an electron-irradiation experiment and observed that the diameters of the smallest and most observed cages were from 0.4 to 0.7 nm, close to those of octahedron-like structures of B_12_N_12_, B_16_N_16_, and B_28_N_28_ cages, which were predicted to be stable magic number clusters by electronic structural calculations [11]. These studies provided experimental evidence for the stable existence of boron nitride clusters.

In fact, early theoretical studies proved that the hollow cage clusters (e.g., B_12_N_12_ [12], B_16_N_16_ [13,14], B_24_N_24_ [15], and B_36_N_36_ [16]) are quite stable. For the specific atomic structure, Strout [17] compared the two classes of boron nitrides: fullerene-like structures consisting of pentagons and hexagons and alternant structures consisting of squares and hexagons. These two classes were compared for B_13_N_13_, B_14_N_14_, and B_16_N_16_ by theoretical calculations using the Hartree–Fock theory and the density functional theory (B3LYP and LDA). The major result was that the alternative structures were more stable than the fullerene-based cage structure.

Over the past few decades, the bottom-up approach, with stable clusters as building blocks, has been considered a promising way to design new materials with desired properties [20,21]. Xiong et al. [22] studied the stabilities and electronic structures of two boron nitride crystals, Pm3n B_12_N_12_ and sc-B_12_N_12_, assembled from the experimental synthesized B_12_N_12_ cluster with an alternative structure by first-principles calculations. They found that the two structures were stable, and both of them were wide-band gap insulators. In our recent previous work, we established a new *sp*^3^-hybridized BN allotrope sc-B_24_N_24_, based on the cage-like alternative B_24_N_24_ cluster, which was energetically, dynamically, and mechanically stable. The analysis of the electronic and optical properties showed that sc-B_24_N_24_ was a semiconductor. Remarkably, if the sc-B_24_N_24_ framework was taken as the host for endohedral doping of magnetic impurities, a desirable magnetic material was obtained, which exhibited a ferromagnetic (FM) half-metallic ground state with complete spin polarization [5]. In all these reported BN cluster-assembled phases, the clusters could maintain their structural characteristics when interacting with neighboring clusters. This means that the clusters B_12_N_12_ and B_24_B_24_ can be served as stable assembly motifs to construct new nanoscale materials using a bottom-up way.

In 2002, Alexandre et al. [23], taking the stable stoichiometric B_16_N_16_ [14] as building blocks, proposed that B_16_N_16_ could form covalent-bound, low-density, cluster-assembled solids with large interstitial channels. However, they only considered one B_16_N_16_-assembled phase. Were the other assembled structures also stable, similar to that of the Zn_16_O_16_ cluster [24]? If so, what about their mechanical and electronic properties?

Inspired by these questions, in this work, based on the density functional theory (DFT), we selected the stable B_16_N_16_ nanocluster as the building block for constructing new possible stable structures. Our results showed that three new porous nanostructures named Cub-B_16_N_16_, Tet-B_16_N_16_, and Ort-B_16_N_16_ were mechanically, dynamically, and thermally stable. On the basis of this, we further explored their structural, mechanical, and electronic properties. As for mechanical properties, the bulk elastic modulus (*B*), shear modulus (*G*), Young’s modulus (*Y*), Pugh’s ratio (*K = B/G*), and elastic anisotropy index (*A^U^*) were calculated. The results of Pugh’s ratio suggested that Cub-B_16_N_16_ and Tet-B_16_N_16_ were brittle materials, but Ort-B_16_N_16_ was ductile. The obtained elastic anisotropy indices indicated that the proposed phases were all anisotropic, and among them, Ort-B_16_N_16_ had the highest anisotropy. Moreover, the calculations of electronic band structure and densities of states revealed that these assembled phases were all indirect band gap semiconductors.

## 2. Computational Methods

The local structure optimization and electronic properties calculations of cluster assembly materials were performed using the DFT method in the Vienna ab initio package (VASP) [25]. The Perdew–Burke–Ernzerhof (PBE) form of the generalized gradient functional (GGA) was used to solve the exchange correlation energy [26,27]. The plane-wave base vector was based on the projector-augmented wave (PAW) method [28]. The Grimme method was adopted to correct the Van der Waals interaction [29]. A cutoff energy of 500 eV was used for the plane-wave basis, and the energy and force constant convergences were set to 10^−6^ eV and 10^−3^ eV Å^−1^, respectively. The Monkhorst-pack *k*-point mesh with uniform spacing was adopted in the Brillouin zones, which were 7 × 7 × 7 for the Cub-B_16_N_16_ structure, 6 × 6 × 7 for the Tet-B_16_N_16_ structure, and 7 × 6 × 7 for the Ort-B_16_N_16_ structure. The phonon dispersion spectra and phonon state densities of the systems were calculated by the density-functional perturbation theory implemented in the PHONOPY package [30] to evaluate the dynamic stability. In addition, molecular dynamics simulations using NVT canonical ensemble at a 300 K temperature were performed to investigate the initial decomposition mechanism and thermal stability of the supercells. The time step was set to 1 fs, and the total simulation time was 10 ps. The elastic constant *C_ij_*, the bulk modulus (*B*, the average of *B*_V_, and *B*_R_), and shear modulus (*G*, the average of *G*_V_, and *G*_R_) of the new structures were calculated according to the Voigt–Reuss–Hill (VRH) approximation [31]. Elastic constants were defined by means of the stress–strain method [32,33].

## 3. Results and Discussion

### 3.1. Structural Properties

**Figure 1:**
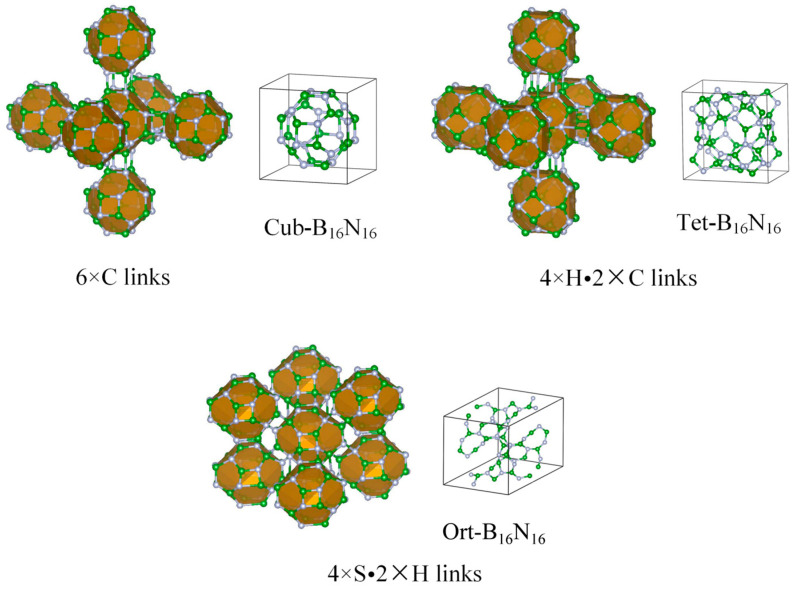
The coordination polymerization mode of each B_16_N_16_ cage in three boron nitride cluster-assembled materials and the primitive cell of the three considered crystal phases. (Cub-B_16_N_16_, Tet-B_16_N_16_, and Ort-B_16_N_16_ were named according to the abbreviation of the crystal system and the name of the construction primitive.)

B_16_N_16_ cage cluster, a stable cluster with magic number characteristics [10], is an octahedral structure, whose basic units are four-membered rings and six-membered rings, among which six four-membered rings are independently separated by twelve six-membered rings [23]. Based on their structural properties and energy stability, we made it the building blocks and considered three possible cluster–cluster interactions, i.e., six-membered ring facing six-membered ring (H), four-membered ring facing four-membered ring (C), and B-N edge-to-edge (S) connection modes. Then, three new periodic 3D solids were obtained and named Cub-B_16_N_16_, Tet-B_16_N_16_, and Ort-B_16_N_16_, according to their crystallographic systems and primitive names. Their optimized atomic structures, including the coordination polymerization mode of each building block and the primitive cells of every phase, are displayed in Figure 1. The Cub-B_16_N_16_, Tet-B_16_N_16_, and Ort-B_16_N_16_ structures had *P*$\bar{4}$3*m*, *P*4/*nbm*, and *Imma* symmetries, respectively.

From Figure 1, one can see that the B_16_N_16_ hollow-cage cluster structures were very stable, with one B atom and four N atoms forming *sp*^3^ hybridization, maintaining its structural integrity in the three assembled new crystalline phases and showing an excellent “element” role. The structural optimization parameters (space groups, lattice constants, unit atomic volumes, and equilibrium densities) of Cub-B_16_N_16_, Tet-B_16_N_16_, and Ort-B_16_N_16_ and several considered structures (e.g., c-BN, d-BN, Hp-BN, Pm3n-BN, and sc-B_12_N_12_) are listed in Table 1. For Cub-B_16_N_16_, Tet-B_16_N_16_, and Ort-B_16_N_16_ structures, the average bond lengths were 1.519 Å, 1.530 Å, and 1.544 Å, respectively; the average bond angles of the four-membered rings were 93.6°, 89.8°, and 90.0°; and the six-membered rings were 119.2°, 117.1°, and 112.8°, respectively. One can see that the mass densities of Cub-B_16_N_16_, Tet-B_16_N_16_, and Ort-B_16_N_16_ were 2.124 g/cm^3^, 2.379 g/cm^3^, and 2.163 g/cm^3^, respectively, which were much lower than that of c-BN (3.472 g/cm^3^) [34], Hp-BN (3.633 g/cm^3^) [35], and Pm3n-BN (2.849 g/cm^3^) [22], due to the existence of hollow holes. The optimized, non-equivalent atomic coordinates of Cub-B_16_N_16_, Tet-B_16_N_16_, and Ort-B_16_N_16_ structures are shown in Table S1. Due to the characteristics of low density and nanopores, the proposed B_16_N_16_-assembled phases might be promising for future applications in heterogeneous catalysis, molecular transport, and other fields [36,37].

### 3.2. Stabilities

**Figure 2:**
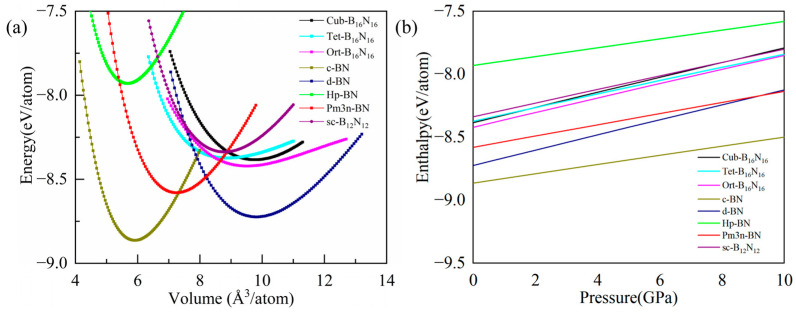
(**a**) The relation curve of total energy as a function of volume per atom for different BN polymorphs. (**b**) The enthalpies varied with pressure per atom for BN cluster-assembled phases, compared with a few BN polymorphs.

Were the Cub-B_16_N_16_, Tet-B_16_N_16_, Ort-B_16_N_16_ polymorphs stable? To explore this, we evaluated their energies and their mechanical, dynamic, and thermal stabilities. Firstly, the total energies of the three assembled materials as functions of volume at a temperature of zero were calculated to determine the energy stability. For comparison, five related boron nitride isomerized materials, including c-BN, d-BN, Hp-BN, Pm3n-BN, and sc-B_12_N_12_ polymorphs, were also considered, as shown in Figure 2a. The results showed that although the equilibrium total energies of these three materials were higher than that of c-BN and d-BN, they were energetically more stable than that of Hp-BN and sc-B_12_N_12_, based on the results, by fitting the third-order Birch–Murnaghan equation of state (EOS) [42]. Among the assembled phases, the Ort-B_16_N_16_ phase had the lowest energy, implying that it was the most stable phase.

The parameters of enthalpy and pressure were obtained from the equation *H* = *E*_t_ + *PV*. The enthalpy pressure relationships of cluster-assembled crystal phases Cub-B_16_N_16_, Tet-B_16_N_16_, and Ort-B_16_N_16_ and a series of boron nitride isomeric phases within the range of 0~10 GPa are shown in Figure 2b to confirm the stabilities of three assembled materials, with respect to the five synthesized phases (e.g., c-BN, d-BN, Hp-BN, Pm3n-BN, and sc-B_12_N_12_) in different ranges of pressure. A more stable phase will generally have a lower enthalpy for a given pressure. It can be seen from Figure 2b that Cub-B_16_N_16_, Tet-B_16_N_16_, and Ort-B_16_N_16_ were all more stable than sc-B_12_N_12_ and Hp-BN in the whole considered ranges of pressure, indicating their good mechanical stabilities with respect to sc-B_12_N_12_ and Hp-BN.

To assess the dynamical stability of the proposed phases, we further calculated the phonon dispersion and the corresponding phonon density of states along highly symmetric paths throughout the Brillouin zone, as shown in Figure S1. The numbers of atoms per unit cell of Cub-B_16_N_16_, Tet-B_16_N_16_, and Ort-B_16_N_16_ were 32, 64 and 64, respectively, indicating that there were 96, 192, and 192 branches of the dispersion spectrum. Since there were no imaginary frequencies in the dispersion spectrum, the proposed Cub-B_16_N_16_, Tet-B_16_N_16_, and Ort-B_16_N_16_ should be dynamically stable at *T* = 0 K. Moreover, the densities of phonon states of the Cub-B_16_N_16_, Tet-B_16_N_16_, and Ort-B_16_N_16_ phases illustrated that the vibrational modes in the low-frequency region were mainly contributed by N atoms, while those in the higher-frequency region were mainly contributed by B atoms, due to its relatively smaller atomic mass.

However, the above discussion could not guarantee the stabilities of the three phases at elevated temperatures. In this regard, further exploration of the thermal stabilities of Cub-B_16_N_16_, Tet-B_16_N_16_, and Ort-B_16_N_16_ at room temperature was necessary. By building a 2 × 2 × 1 supercell with 128 atoms for the Cub-B_16_N_16_ structure, 256 atoms for the Tet-B_16_N_16_ structure, and a 2 × 1 × 1 supercell with 128 atoms for the Ort-B_16_N_16_ structure, we performed the ab initio molecular dynamics (AIMD) simulations at 300 K with a Nosé–Hoover thermostat. Figure S2 shows the potential energy and temperature fluctuations of the three systems as a function of simulation times. Throughout the simulation, the potential energy was almost constant, with small variations due to thermal fluctuations for all the assembled crystal phases. Correspondingly, the structures maintained their original structures without damage. Therefore, the simulation results confirmed that all the assembled structures were thermally stable and could survive, at least at room temperature.

To examine the mechanical stability of the assembled phases, we further calculated their elastic constants. The results are listed in Table 2. According to Born’s mechanical stability criterion, a material with mechanical stability should follow the related mechanical stability criteria:

For the cubic system,

(1) $$C_{11}>0,C_{44}>0,C_{11}-C_{12}>0,C_{11}+2C_{12}>0.$$

For the tetragonal system,

(2) $$C_{11}>0,C_{33}>0,C_{44}>0,C_{66}>0,\;C_{11}-C_{12}>0,C_{11}+C_{33}–2C_{13}>0,\;2(C_{11}+C_{12})+C_{33}+4C_{13}>0.$$

For the orthorhombic system,

(3) $$C_{11}>0,C_{22}>0,C_{33}>0,C_{44}>0,C_{55}>0,C_{66}>0,\;C_{11}+C_{22}-2C_{12}>0,C_{11}+C_{33}-2C_{13}>0,C_{22}+C_{33}-2C_{23}>0,\;C_{11}+C_{22}+C_{33}+2\left(C_{12}+C_{13}+C_{23}\right)>0.$$

It can be seen that all of the *C_ij_* for Cub-B_16_N_16_, Tet-B_16_N_16_, and Ort-B_16_N_16_ met the Born stability criterion [43]; this means that they were all mechanically stable.

**Figure 3:**
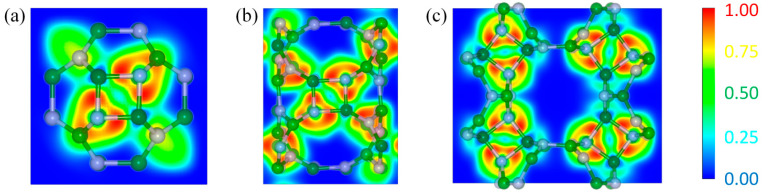
The 2D contour plots of the electron localization functions (ELF) of (**a**) Cub-B_16_N_16_, (**b**) Tet-B_16_N_16_, and (**c**) Ort-B_16_N_16_ along the [001] direction. The reference bar for the ELF value is provided on the right.

Electron localization function (ELF) was an effective method for analyzing the types of chemical bonds, which can accurately characterize the distribution characteristics of electron delocalization in both molecules and solids [45]. Values of 1.00 and 0.50 indicated complete localization and delocalization of electrons, respectively, while 0.00 indicated very low electron density. The electronic local functions of Cub-B_16_N_16_, Tet-B_16_N_16_, and Ort-B_16_N_16_ along the four-numbered ring in the [001] direction was calculated to investigate the local characteristics of the assembled materials. Figure 3 illustrates a 2D contour map of ELF along the [001] direction of Cub-B_16_N_16_, Tet-B_16_N_16_, and Ort-B_16_N_16_. It can be seen that the ELF value in the middle of the B-N bond was close to 1.0, implying that the electrons were highly localized in this region. In other words, the B-N bonds in the assembled materials had strong covalent properties. This should be the reason that Cub-B_16_N_16_, Tet-B_16_N_16_, and Ort-B_16_N_16_ had superb energy and dynamical, thermal, and mechanical stability. Near the N and B atoms, the ELF values were about 0.5 and 0.25, respectively. This means that electrons were more likely to be localized around the N atom, while the densities of electrons near the B atom were very low.

### 3.3. Mechanical Properties

As is known, many reported polymorphs of boron nitride have excellent mechanical properties. This naturally poses a question: can the new low-density porous boron nitride polymorphs preserve their intrinsic configuration under external stress? Using the VRH method [31], we calculated the bulk elastic modulus (*B*), shear modulus (*G*), Young’s modulus (*Y*), and Pugh’s ratio (*K = B/G*) of the three low-density assembled phases on the basis of the obtained elastic constants. The data are listed in Table 2. The moduli of Cub-B_16_N_16_, Tet-B_16_N_16_, and Ort-B_16_N_16_ were relatively lower, compared to that of the super-hard boron nitride c-BN. However, considering their low densities, they still had good elastic properties. To prove this, we further calculated the Vickers hardness (*H_v_*) using the empirical formula [37]:

(4) $$H_{\nu }=2{\left(\frac{G^{2}}{B^{2}}\right)}^{0.585}-3$$

The calculated results of Vickers hardness are shown in Table 2. One can see that the hardnesses of Cub-B_16_N_16_, Tet-B_16_N_16_, and Ort-B_16_N_16_ were less than 40 GPa, which was the critical value of Vickers hardness to distinguish super-hard materials from ordinary materials. Although they were not super-hard materials, Cub-B_16_N_16_, Tet-B_16_N_16_, and Ort-B_16_N_16_ were still hard, even superior to some metal nitrides and carbides, such as TiC, TiN, and WC [46]. In addition, the ratio of the bulk elastic modulus to the shear modulus (i.e., *B*/*G*) is often used to distinguish ductile and brittle materials. If the *B*/*G* ratio is greater than 1.75, the material is ductile; otherwise, it is brittle. The *B*/*G* ratios of Cub-B_16_N_16_, Tet-B_16_N_16_, and Ort-B_16_N_16_ were calculated to be 1.61, 1.60, and 2.07, respectively. There was no doubt that Cub-B_16_N_16_ and Tet-B_16_N_16_ were brittle materials, but Ort-B_16_N_16_ was ductile.

**Figure 4:**
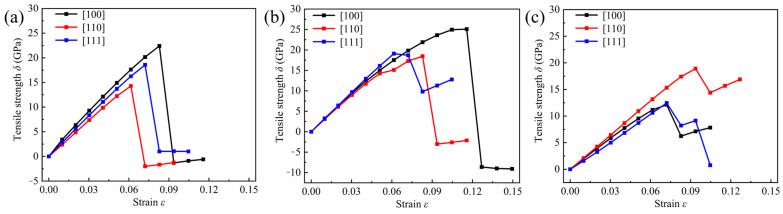
Ideal tensile strength along the [100], [110], and [111] directions for (**a**) Cub-B_16_N_16_, (**b**) Tet-B_16_N_16_, and (**c**) Ort-B_16_N_16_.

Figure 4 shows the ideal tensile strength as a function of strain for Cub-B_16_N_16_, Tet-B_16_N_16_, and Ort-B_16_N_16_ along the three directions of [100], [110], and [111]. It can be seen that Cub-B_16_N_16_, Tet-B_16_N_16_, and Ort-B_16_N_16_ could withstand a certain tensile strain before the structure broke. Specifically, along the [100], [110], and [111] directions, the ideal tensile strength and the corresponding maximum strain withstood at the fracture point of the three structures are shown in Table 3.

Moreover, from Figure 4, one can conclude that Cub-B_16_N_16_, Tet-B_16_N_16_, and Ort-B_16_N_16_ were mechanically anisotropic. Microcracks and lattice deformations of materials are important factors for reflecting the elastic anisotropy, which also plays a key role in enhancing the mechanical durability of materials. In order to characterize the anisotropy degree of materials, Ranganathan proposed a universal elastic anisotropy index *A^U^* for each crystal phase, based on the mean values of Reuss and Voigt [47]:

(5) $$A^{U}=5\frac{G_{V}}{G_{R}}+\frac{B_{V}}{B_{R}}-6$$

where *G_V_*, *G_R_*, *B_V_*, and *B_R_* are the shear moduli and bulk moduli of Voigt and Reuss approximations, respectively.

**Figure 5:**
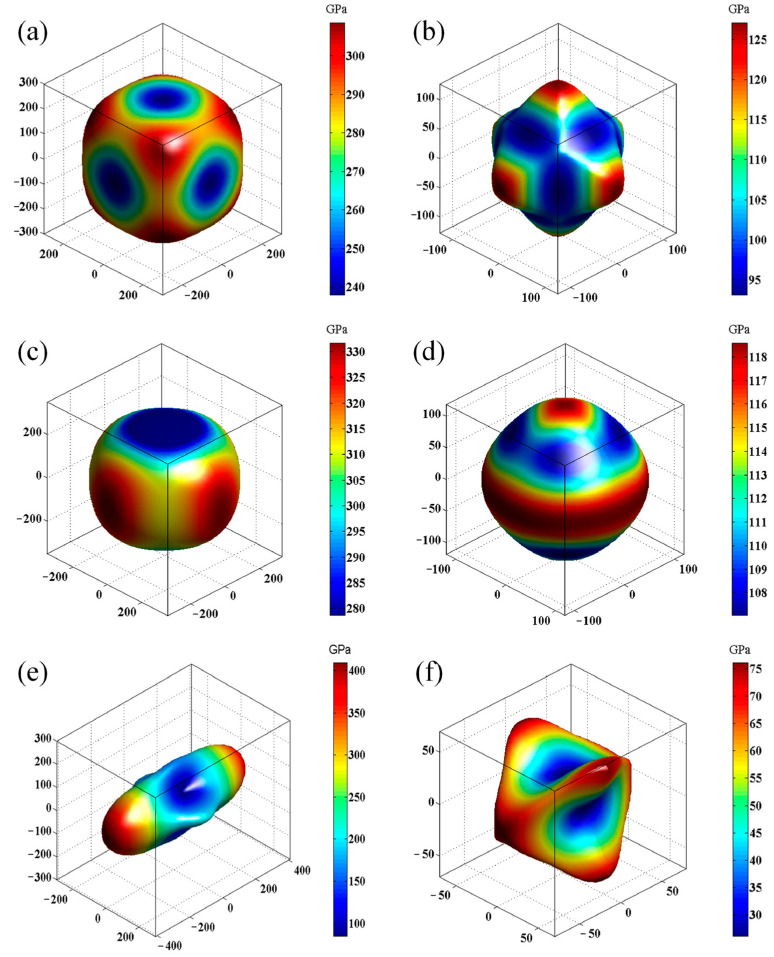
The directional dependence of Young’s modulus (*Y*) for (**a**) Cub-B_16_N_16_, (**c**) Tet-B_16_N_16_, and (**e**) Ort-B_16_N_16_ and shear modulus (*G*) for (**b**) Cub-B_16_N_16_, (**d**) Tet-B_16_N_16_, and (**f**) Ort-B_16_N_16_.

The material is isotropic when the value of *A^U^* is zero; otherwise, it is anisotropic. The degree of anisotropy of the material can be reflected by the magnitude of the *A^U^* deviation from zero. The greater the deviation of *A^U^*, the stronger the anisotropy. We calculated the universal elastic anisotropy indices, *A^U^*, of Cub-B_16_N_16_, Tet-B_16_N_16_, and Ort-B_16_N_16_ to provide an effective perspective on mechanical anisotropy. The results showed that the anisotropy indices of Young’s moduli for Cub-B_16_N_16_, Tet-B_16_N_16_, and Ort-B_16_N_16_ were 1.30, 1.32, and 4.82, respectively, and the anisotropy indices of shear moduli were 1.37, 1.39, and 4.62, respectively. Therefore, Cub-B_16_N_16_, Tet-B_16_N_16_, and Ort-B_16_N_16_ were all anisotropic. Among the proposed phases, Ort-B_16_N_16_ had the strongest anisotropy, due to its largest *A^U^*. Figure 5 shows the three-dimensional diagrams of Young’s moduli and shear moduli, which can provide more intuitive physical images for the anisotropic elastic characteristics of the three assembly structures.

### 3.4. Electronic Properties

**Figure 6:**
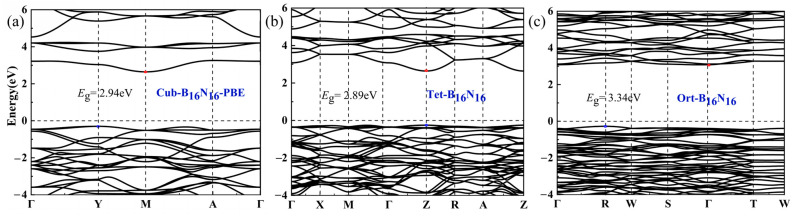
Graphs (**a**–**c**) are the electronic band structures of Cub-B_16_N_16_, Tet-B_16_N_16_, and Ort-B_16_N_16_, respectively.

The electronic band structures along the high-symmetry *k*-points were calculated, and the Monkhorst-pack *k*-point meshes increased to 15 × 15 × 15 for the Cub-B_16_N_16_ structure, 11 × 11 × 15 for the Tet-B_16_N_16_ structure, and 12 × 15 × 14 for the Ort-B_16_N_16_ structure, as shown in Figure 6. Similar to most of the BN polymorphs, one can see that Cub-B_16_N_16_, Tet-B_16_N_16_, and Ort-B_16_N_16_ were semiconducting with wide energy band gaps, which were 2.94, 2.80, and 3.34 eV, respectively. Since the valence band maximum (VBM) and conduction band minimum (CBM) were not at the same high-symmetry *k*-points for Cub-B_16_N_16_ and Ort-B_16_N_16_ structures, they belonged to indirect band gap semiconductors. However, the Tet-B_16_N_16_ structure was a direct bandgap semiconductor, since VBM and CBM were at the same high-symmetry *k*-points. As is usual for PBE calculations, the absolute band gaps were systematically underestimated, whereas the relative magnitudes provided a gauge of the electronic change with respect to the change in bulk topology [48]. We calculated the relative band gap magnitude of each structure to the most stable c-BN (*E*_g_/*E*_g,c_), which is shown in Table 1.

**Figure 7:**
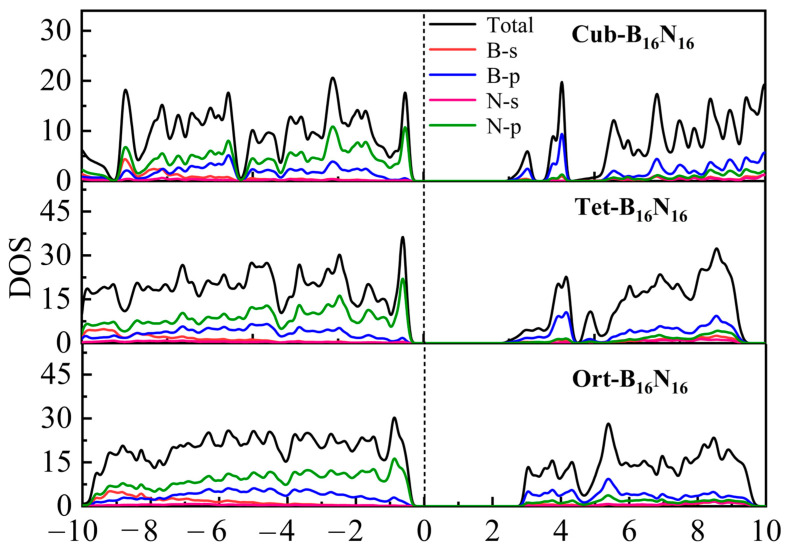
Total and partial DOS for Cub-B_16_N_16_, Tet-B_16_N_16,_ and Ort-B_16_N_16_ at equilibrium structure.

The total and partial densities of states (DOS) of Cub-B_16_N_16_, Tet-B_16_N_16_, and Ort-B_16_N_16_ are shown in Figure 7. There was obvious hybridization between the N atomic orbital and the B orbital, due to the fact that the B-N bonds of the assembled crystal phases were strong covalent bonds. According to the partial densities of states, the densities of states of the VBM and CBM were dominated by B-2*p* and N-2*p* orbitals, respectively. In the vicinity of the Fermi level, the states of the valence were mainly contributed by the N-2*p* orbital, and the states of the conduction band mainly came from the B-2*p* orbital.

## 4. Conclusions

In summary, based on the bottom-up approach, we predicted three new low-density boron nitride polymorphs, Cub-B_16_N_16_, Tet-B_16_N_16_. and Ort-B_16_N_16_, which can be considered three-dimensional structures assembled from B_16_N_16_ cage clusters. Based on the density functional theory modified by Van der Waals, the following interesting features of Cub-B_16_N_16_, Tet-B_16_N_16_, and Ort-B_16_N_16_ were characterized: (i) they were low-density (2.124 g/cm^3^, 2.379 g/cm^3^, and 2.163 g/cm^3^, respectively) porous materials, due to the existence of boron nitride hollow cages B_16_N_16_; (ii) Cub-B_16_N_16_, Tet-B_16_N_16_, and Ort-B_16_N_16_ exhibited good energy and dynamic, thermal, mechanical, and chemical stability, due to the strong covalence interaction between B and N atoms, which was proven by our electron localization function analysis; (iii) the results of elastic properties calculations showed that Cub-B_16_N_16_ and Tet-B_16_N_16_ were brittle materials, but Ort-B_16_N_16_ was ductile; the Young’s moduli and shear moduli of the three assembled materials harbored strong anisotropy; (iv) the electronic band structure showed that the three assembled crystal phases were all indirect wide-band gap semiconductors. Our results not only highlighted some novel low-density boron nitride polymorphs, they also provided a bottom-up way to design new solid materials by using the clusters as building blocks.

## Figures and Tables

**Table 1:** The space group (*SG*), lattice parameters *a*, *b*, and *c* (Å), volume per atom *V* (Å^3^/atom), equilibrium density *ρ* (g/cm^3^), cohesive energy per atom *E*_tot_ (eV/atom), energy gap *E_g_* (eV), and band gap relative to ground-state c-BN phase (*E_g_/E_g_*_,c_) for cluster-assembled BN phases. The corresponding data of several reported BN polymorphs (c-BN, 2D h-BN, w-BN, d-BN, Hp-BN, Pm3n-BN, and sc-B_12_N_12_) were also compared.

Structure		*SG*	*a*(Å)	*b*(Å)	*c*(Å)	*V*	*ρ*	*E*_tot_(eV)	*E_g_*(eV)	*E_g_/E_g_*_,c_
Cub-B_16_N_16_		$P\bar{4}3m$	6.772	6.772	6.772	9.77	2.124	−8.38	2.94	0.659
Tet-B_16_N_16_		*P*4/*nbm*	8.991	8.991	6.857	8.89	2.379	−8.37	2.80	0.628
Ort-B_16_N_16_		*Imma*	8.975	12.669	10.726	9.51	2.163	−8.42	3.34	0.749
c-BN	This work	$F\bar{4}3m$	3.615	3.615	3.615	5.90	3.489	−8.86	4.46	1.00
	Cal. [22]		3.625	3.625	3.625			−9.37		
	Expt. [38]		3.615	3.615	3.615		3.489		6.1~6.4	
h-BN(2D)	This work	*P*6_3_/*mmc*	2.506	2.506				−8.16	3.95	0.89
	Cal. [39]		2.512	2.512						
	Expt. [40]		2.490	2.490						
w-BN	This work	*P*63*mc*	2.549	2.549	4.231	5.92	2.095	−8.85	5.20	1.17
	Cal. [22]		2.555	2.555	4.225			−9.35		
	Expt. [41]		2.553	2.553	4.228					
d-BN	This work	$F\bar{d}3c$	12.290	12.290	12.290	9.81	2.101	−8.72	4.84	1.09
	Cal. [36]		12.292	12.292	12.292		2.130		4.86	
Hp-BN	This work	*P*6_2_22	2.600	2.600	5.811	5.67	3.633	−7.93	3.70	0.83
	Cal. [35]		2.610	2.610	5.828			−7.78	3.45	
Pm3n-BN	This work	$Pm\bar{3}n$	4.428	4.428	4.428	7.23	2.849	−8.58	4.55	1.02
	Cal. [22]		4.418	4.418	4.418		2.868	−8.33	4.53	
sc-B_12_N_12_	This work	$Fm\bar{3}C$	11.819	11.819	11.819	8.75	2.345	−8.33	4.98	1.12
	Cal. [22]		11.819	11.819	11.819		2.396	−8.20	5.02	

**Table 2:** Calculated elastic constants *C_ij_* (GPa), bulk modulus *B* (GPa), shear modulus *G* (GPa), Pugh’s ratio *K* (*B/G*), Young’s modulus *Y* (GPa), and Vickers hardness *H_v_* (GPa) of cluster-assembled BN phases and a few typical BN phases.

Structure	*C*_11_	*C*_12_	*C*_13_	*C*_22_	*C*_23_	*C*_33_	*C*_44_	*C*_55_	*C*_66_	*B*	*G*	*K*	*Y*	*H_v_*
Cub-B16N16	304	118					127			180	112	1.61	279	15.19
Tet-B16N16	376	84	122			314	123		134	197	123	1.60	305	16.30
Ort-B16N16	225	73	54	480	112	200	76	26	76	141	69	2.07	178	8.17
c-BN	797	175					456			382	391	0.98	875	63.37
Cal. [44]	780	173					444			376	382			62.82
d-BN	300	175					120			216	93	2.32	243	7.38
Cal. [36]										252	111	2.17		
Hp-BN	873	154					360			384	366	1.04	832	55.97
Cal. [35]	892	166					363			375				
Pm3n-BN	712	90					195			297	235	1.26	558	33.74
Cal. [22]	781	116					218			337	218~332			
sc-B12N12	452	127					163			232	162	0.69	391	17.28
Cal. [22]	483	160					190			268	162~190			

**Table 3:** Calculated ideal tensile strengths and corresponding maximum tensile strains withstood at the fracture point.

Structure	Direction	Ideal Tensile Strength (GPa)	Maximum Strain (%)
Cub-B_16_N_16_	[100]	22.39	8
	[110]	14.29	6
	[111]	18.56	7
Tet-B_16_N_16_	[100]	25.09	12
	[110]	18.47	8
	[111]	18.69	7
Ort-B_16_N_16_	[100]	12.12	7
	[110]	18.91	9
	[111]	12.45	7

## Data Availability

The data presented in this study are available upon request from the corresponding author.

## Supplementary Materials

The following supporting information can be downloaded at: https://www.mdpi.com/article/10.3390/nano13131927/s1, Table S1: The optimized non-equivalent atomic coordinates of Cub-B_16_N_16_, Tet-B_16_N_16_, and Ort-B_16_N_16_ structures. Figure S1: Phonon band structures and phonon density of states of Cub-B_16_N_16_, Tet-B_16_N_16_, and Ort-B_16_N_16_. Figure S2: The fluctuation of potential energy and temperature of Cub-B_16_N_16_, Tet-B_16_N_16_, and Ort-B_16_N_16_ as a function of molecular dynamics simulation time at room temperature. Reference [49] is cited in the Supplementary Materials.
