# Supplementary material for: First-Principles Study of B16N16 Cluster-Assembled Porous Nanomaterials
Source: Nanomaterials (Basel). 2023 Jun 24;13(13):1927. doi: 10.3390/nano13131927 (PMC10343589; doi:10.3390/nano13131927)
Supplement: Supplementary file 1 [file nanomaterials-13-01927-s001.zip › nanomaterials-2451898-supplementary.pdf]

nanomaterials

Supplementary Materials

First-Principles Study of $B_{16}N_{16}$ Cluster-Assembled Porous Nanomaterials

Xin Wang, Xiaoyue Zhang, Liwei Liu, Tielei Song, Zhifeng Liu and Xin Cui *

School of Physical Science and Technology, Inner Mongolia University, Hohhot 010021, China;
* Correspondence: pycuixin@imu.edu.cn

1. Structure Parameter

The non-equivalent atomic coordinates of Cub-$B_{16}N_{16}$, Tet-$B_{16}N_{16}$, and Ort-$B_{16}N_{16}$ structures are shown in Table S1.

Table S1. The optimized, non-equivalent atomic coordinates of Cub-$B_{16}N_{16}$, Tet-$B_{16}N_{16}$, and Ort-$B_{16}N_{16}$ structures.

atom	Cub-$B_{16}N_{16}$			Tet-$B_{16}N_{16}$			Ort-$B_{16}N_{16}$		
	x	*y*	*z*	*x*	*y*	*z*	*x*	*y*	*z*
N_1	0.378	0.622	0.120	0.586	0.707	0.868	0.854	0.056	0.375
N_2	0.725	0.275	0.725	0.848	0.652	0.730	1.000	0.335	0.224
N_3				0.093	0.593	0.614	0.730	0.250	0.138
N_4							0.775	0.146	0.570
N_5							0.874	0.250	0.767
N_6							1.000	0.087	0.688
B_1	0.615	0.385	0.880	0.713	0.586	0.887	1.000	0.087	0.314
B_2	0.280	0.721	0.280	0.835	0.665	0.226	0.860	0.055	0.623
B_3				0.918	0.582	0.624	1.000	0.177	0.756
B_4							0.787	0.148	0.438
B_5							0.879	0.250	0.230
B_6							0.702	0.250	0.644

2. Phonon spectrum and molecular dynamic (AIMD) simulations

The phonon dispersion and the corresponding phonon density of states along highly symmetric paths throughout the Brillouin zone are shown in Figure S1. The numbers of atoms per unit cell of Cub-$B_{16}N_{16}$, Tet-$B_{16}N_{16}$, and Ort-$B_{16}N_{16}$ were 32, 64, and 64, respectively, indicating that there were 96, 192, and 192 branches of the dispersion spectrum. The ab initio molecular dynamics (AIMD) simulations at 300 K with a Nosé–Hoover thermostat are shown in Figure S2.

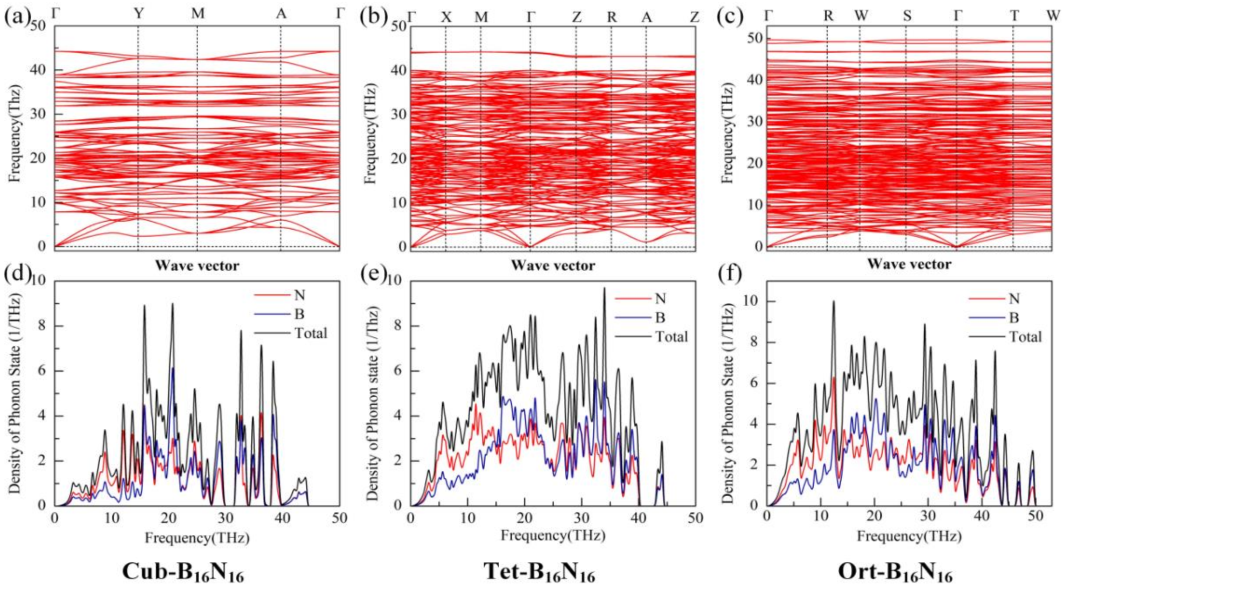

Figure S1. Phonon band structures and phonon densities of states of Cub-$B_{16}N_{16}$, Tet-$B_{16}N_{16}$, and Ort-$B_{16}N_{16}$.

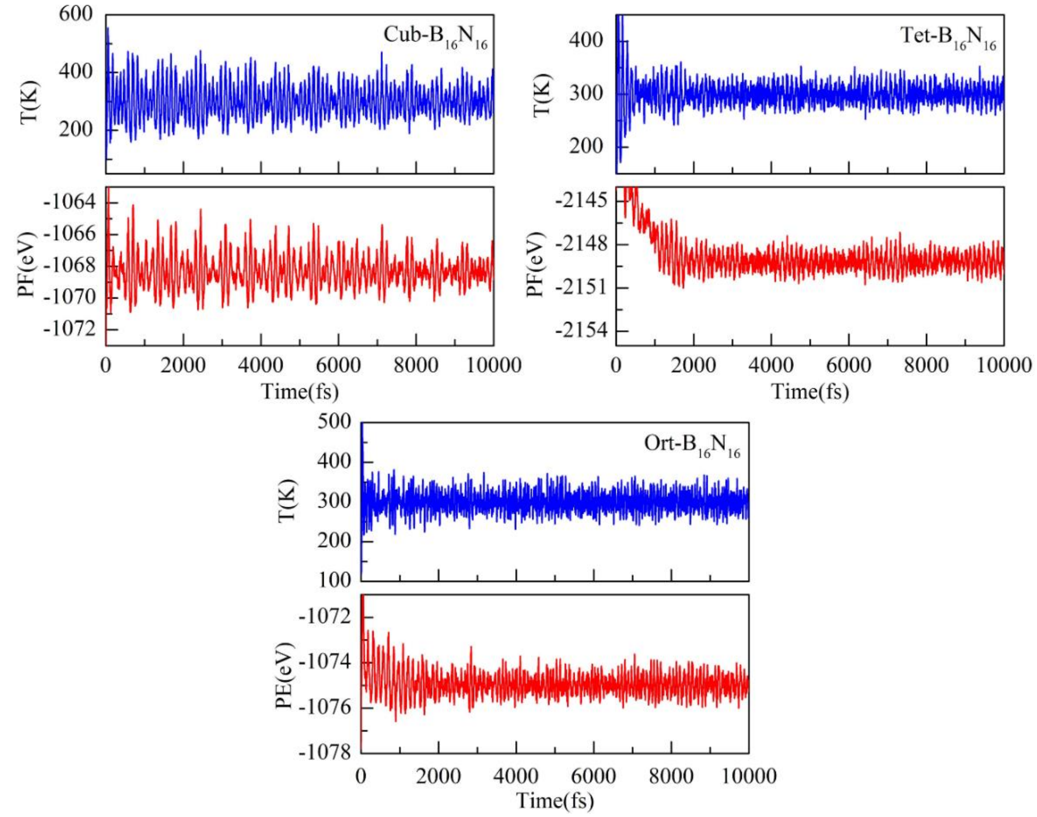

Figure S2. The fluctuation of potential energy and temperature of Cub-$B_{16}N_{16}$, Tet-$B_{16}N_{16}$, and Ort-$B_{16}N_{16}$ as a function of molecular dynamics simulation time at room temperature.

3. Elastic constants and elastic moduli

Elastic constants are defined by means of the stress–strain method [32,33]. The exact formula is given as:

$$\sigma_{ij} = C_{ijkl}\varepsilon_{kl} \tag{1}$$

where σ_{ij} is the stress tensor, ε_{kl} is the Lagrangian strain tensor, and C_{ijkl} is the elastic constant tensor, which is a 6×6 matrix (36 elements in general cases).

We made use of the contracted Voigt notation [49] 11→1, 22→2, 33→3, 23→4, 13→5, and 12→6 for the tensor indices to write C_{ijkl} as $C_{\alpha\beta}$. The independent elements of the elastic constant tensor depended on the crystal symmetry. There were three independent constants for the cubic phase, which were C_{11}, C_{12}, and C_{44}; six independent constants for tetragonal symmetry, namely C_{11}, C_{12}, C_{13}, C_{33}, C_{44}, and C_{66}; and nine independent constants for the orthorhombic phase, i.e., C_{11}, C_{12}, C_{13}, C_{22}, C_{23}, C_{33}, C_{44}, C_{55}, and C_{66}.

The Voigt (B_V) and Reuss (B_R) bulk moduli, the Voigt (G_V) and Reuss (G_R) shear moduli, and Young's modulus (Y) were calculated using the following equations:

For the cubic system,

$$B_V = B_R = \frac{1}{3}(C_{11} + 2C_{12}) \tag{2}$$

$$G_V = \frac{1}{5}(C_{11} - C_{12} + 3C_{44}) \tag{3}$$

and

$$G_R = \frac{5C_{44}(C_{11} - C_{12})}{4C_{44} + 3(C_{11} - C_{12})} \tag{4}$$

For the tetragonal system,

$$B_V = \frac{2(C_{11} + C_{12}) + 4C_{13} + C_{33}}{9} \tag{5}$$

$$B_R = \frac{C_{33}(C_{11} + C_{12}) - 2C_{13}^2}{C_{11} + C_{12} + 2C_{33} - 4C_{13}} \tag{6}$$

$$G_V = \frac{12(C_{44} + C_{66}) + C_{11} + C_{12} + 2C_{33} - 4C_{13}}{30} \tag{7}$$

and

$$G_R = \frac{5C_{44}(C_{11} - C_{12})}{4C_{44} + 3(C_{11} - C_{12})} \tag{8}$$

For the orthorhombic system,

$$B_V = \frac{1}{9}(C_{11} + C_{22} + C_{33}) + \frac{2}{9}(C_{12} + C_{13} + C_{23}) \tag{9}$$

$$B_R = \left[\left(S_{11} + S_{22} + S_{33}\right) + 2\left(S_{12} + S_{13} + S_{23}\right)\right]^{-1} \tag{10}$$

$$G_V = \frac{1}{15}(C_{11} + C_{22} + C_{33} - C_{12} - C_{13} - C_{23}) + \frac{1}{5}(C_{44} + C_{55} + C_{66}) \quad (11)$$

and

$$G_R = 15\left[4\left(S_{11} + S_{22} + S_{33}\right) - 4\left(S_{12} + S_{13} + S_{23}\right) + 3\left(S_{44} + S_{55} + S_{66}\right)\right]^{-1} \quad (12)$$

where S_{ij} is the inverse matrix of the elastic constant matrix C_{ij}.

Young's modulus was obtained using the following equation:

$$Y = \frac{9BG}{3B + G} \quad (13)$$

Reference

49. Carrasco, J.; Illas, F.; Bromley, S.T. Ultralow-density nanocage-based metal-oxide polymorphs. *Phys. Rev. Lett.* **2007**, *99*, 235502.
